# Lateral supraorbital approach applied to sellar tumors in 23 consecutive patients: the Suzhou experience from China

**DOI:** 10.1186/1477-7819-11-41

**Published:** 2013-02-21

**Authors:** Gang Chen, Zhong Wang, Dai Zhou

**Affiliations:** 1 Department of Neurosurgery, The First Affiliated Hospital of Soochow University, 188 Shizi Street, Suzhou, Jiangsu Province, 215006, China

**Keywords:** Lateral supraorbital approach, Pituitary adenoma, Craniopharyngioma, Meningioma

## Abstract

**Background:**

Pituitary adenoma, craniopharyngioma and meningioma are common sellar lesions, accounting for more than 90% of sellar tumors. The aim of this study is to assess the reliability and safety of the lateral supraorbital (LSO) approach to remove sellar tumors.

**Methods:**

Between June 2011 and December 2011, 23 patients with neoplastic lesions underwent surgery by the corresponding author (ZW) via the LSO approach. The clinical presentations, neuroradiological findings, microsurgical techniques, and outcome at discharge of these patients were analyzed. In addition, the clinical series in the available literature written in English were also extensively reviewed. Eleven (48%) patients were treated for pituitary adenoma; six (26%) patients for tuberculum sellae meningioma; and six (26%) patients for craniopharyngioma.

**Results:**

Seemingly complete tumor removal was achieved in 21 patients (91%); surgical mortality was one patient (4.3%). Postoperatively, no patient had developed cerebrospinal fluid leakage or new visual deficits. One (4%) patient had intracranial infection, and one (4%) had a postoperative hematoma. The median Karnofsky score at discharge was 87.4 (range, 0 to 100). The Glasgow outcome scale at discharge was 4.6 (range, 1 to 5). Of all, 21 (91.3%) patients achieved favorable outcomes.

**Conclusion:**

Sellar tumors can be removed via the LSO approach with relatively low morbidity and mortality. Surgical results with this fast and simple approach are similar to those obtained with more extensive, complex, and time-consuming approaches.

## Background

Pituitary adenomas, craniopharyngiomas and tuberculum sellae meningioma, are by far the most common tumors of the sellar region, comprising 90% of all such tumors [1]. The majority of pituitary adenomas are asymptomatic, discovered as ‘incidentalomas’ in the course of investigation for other conditions. The remainder, along with other sellar tumors, present with symptoms of endocrine disorder, mass effect on adjacent structures - usually the optic nerves or chiasm, or headache [2]. Meningiomas of the sellar region consist of approximately 14% of the intracranial total, occurring on the tuberculum sella or planum sphenoidale, on the medial sphenoid wing and cavernous sinus [3]. Craniopharyngiomas comprise approximately 3% of all intracranial tumors. Although half of these occur in adults, they account for a greater percentage of childhood tumors (5 to 13%) and are responsible for 54% of sellar region pathology in children. There are important differences in clinical presentation, pathology and outcome between children and adults [4].

Although the standard pterional approach is considered the gold standard neurosurgical route to reach the sellar area, this approach requires manipulation of the temporalis muscle. This maneuver may be associated with significant atrophy of the muscle as well as with different degrees of dysfunction of the frontal branch of the facial nerve [5]. Several surgical modifications have been suggested to reduce this potential problem. The lateral supraorbital (LSO) approach described by Hernesniemi *et al*. is one such alternative approach. Hernesniemi *et al*. propose this approach to operate on intracranial lesions located in the sellar and suprasellar regions, in the Sylvian fissure, and in the retrosellar regions such as the superior part of the basilar artery and the interpeduncular fossa [6,7]. This modified technique has the advantages of requiring a short skin incision that does not reach the front of the ear, unlike the incision when using the standard pterional approach, thus causing less trauma to the temporalis muscle. Additionally, there is no risk of injury to the upper branch of the facial nerve because the use of a myocutaneous flap protects this nerve. This incision also has an excellent cosmetic result because it is usually hidden behind the hair line [8].

However, until now there has been no clinical report published concerning the LSO approach applied to pituitary adenoma and craniopharyngioma, and also no study was found in the literature on the LSO approach and sellar tumors in China. For this article, we reviewed the characteristics and outcome of 23 patients with sellar lesions operated on by the senior author (ZW) using the LSO approach between June 2011 and December 2011 at the Department of Neurosurgery of the First Affiliated Hospital of Soochow University, China. We demonstrate that these tumors can be removed by using the simple and fast LSO approach, with results comparable to those obtained using more extensive surgical approaches.

## Methods

Twenty-three patients with sellar tumors were treated by lateral supraorbital (LSO) procedures. All operations were performed by the same surgeon (ZW) at the Department of Neurosurgery of the First Affiliated Hospital of Soochow University, China. Patients’ medical records, clinical visits, and imaging studies were reviewed and data on tumor characteristics, intraoperative and postoperative complications, and surgical outcomes of patients were collected. All patients had at least a six-month follow-up clinic visit and computed tomography (CT) scans. Patients with tumors approached by a conventional frontotemporal route or endonasal route were not included in this analysis. This retrospective study was approved by Soochow University.

Patient files and radiological data were reviewed. The demographics and clinical findings are presented in Table 1. Of the 23 patients, 12 (52%) were women and 11 (48%) were men, with a median age of 53 years (range, 4 to 78 years). The preoperative and postoperative clinical conditions were expressed by the Karnofsky performance scale [9], and the Glasgow outcome scale [10] was used to reflect the postoperative clinical outcome.

**Table 1:** Summary of our cases

**Case number**	**Age/Sex**	**Diagnosis**	**Maximum tumor diameter (mm)**	**Tumor location**	**Prior surgery**	**Cavernous sinus invasion/vascular encasement**	**Extent of resection**	**Visual changes**	**GOS at discharge**	**Karnofsky score at discharge**	**Follow-up (months)**
1	70/F	PA	31	S, SS	0	0	Total	↑	5	100	17
2	78/M	PA	35	RC, S,	0	Yes	Near	→	4	80	12
				SS			Total				
3	63/M	PA	27	S,SS	0	0	Total	↔	5	100	11
4	69/M	PA	33	S,SS	0	0	Total	↑	5	100	12
5	12/M	PA	26	S,SS	0	0	Total	→	5	100	14
6	48/F	PA	30	RC,S,	0	0	Total	↔	5	100	13
				SS							
7	55/M	PA	40	RC,S	Yes	Yes	Near	↑	4	80	12
				SS			Total				
8	76/M	PA	30	S,SS	Yes	0	Total	↑	4	80	13
9	42/F	PA	42	RC,S	0	Yes	Subtotal	↑	5	100	12
				SS							
10	47/F	PA	36	S,SS	0	0	Total	↔	5	100	13
11	60/M	PA	32	S,SS	0	Yes	Total	↑	5	100	12
12	29/M	CR	40	RC, S	0	Yes	Near	↑	4	80	13
13	31/M	CR	34	RC, S, SS	0	0	Total	↑	5	100	11
14	62/F	CR	36	RC, S, SS, Ext	0	Yes	Near Total	↑	4	80	12
15	45/F	CR	29	S, SS	0	0	Total	→	5	100	12
16	56/F	CR	35	RC, S, SS	0	0	Near Total	↔	5	100	13
17	4/F	CR	23	RC, S, SS	Yes	0	Subtotal	→	5	100	12
18	64/F	TSM	32	SS, Ext	0	Yes	Total	→	5	100	11
19	27/F	TSM	35	SS	0	0	Total	↑	5	100	12
20	43/M	TSM	43	SS	0	Yes	Near Total	→	3	30	11
21	53/M	TSM	32	S, SS	0	0	Total	↑	5	100	14
22	57/F	TSM	39	SS, Ext	0	Yes	Total	→	1	0	0
23	55/F	TSM	36	SS	0	0	Total	↑	5	100	10

Note: PA,pituitary adenoma; CR, craniopharyngioma; TSM, tuberculum sellae meningioma; GOS, Glasgow outcome scale; RC, retrochiasmal; SS, suprasellar; S, sellar; Ext, extensive; ↑, improved postoperative vision; ↓, worsened postoperative vision; →, preoperative visual impairment not improved; ↔, no visual impairment pre- or postoperatively.

The LSO approach is a less invasive modification of the pterional approach, located more frontally and with a bone flap of approximately 3 cm in diameter. It was first created and used by Juha Hernesniemi from Helsinki University Central Hospital for vascular and neoplastic lesions of the anterior cranial base and has been described in detail previously [11].

Briefly, the head fixed to the head frame is 1) elevated well above the level of the heart, 2) rotated 20 to 30 degrees toward the opposite side, and 3) tilted laterally for optimal visualization of the attachments of the tumor. The neck of the patient is slightly flexed to obtain a better view of the anterior part of the sellar region. We adjusted the position of the fixed head and body during the operation as needed [12]. Usually, a small LSO craniotomy is all that is necessary. A single burr hole is placed just under the temporal line of the bone, that is, the superior insertion of the temporal muscle. A 3 cm bone flap is detached mostly by a side-cutting drill, and the basal part can be drilled before lifting. The dura is incised curvilinearly with the base sphenoidally. Dural edges are elevated by multiple stitches extended over the craniotomy dressings. From this point onward, all surgery is performed under the operating microscope.

Slack brain is achieved by both neuroanesthesia and intraoperative release of cerebrospinal fluid (CSF) [13]. The floor of the anterior cranial base is followed toward the ipsilateral optic nerve and carotid artery, and CSF is released from the basal cisterns. In our experience, opening the Sylvian fissure is not necessary but if needed for larger tumors, it can be easily performed via the LSO approach, as reported by previous studies [14]. After opening of the basal cisterns, having achieved a slack brain and more space under the frontal lobe, dissection is directed medially toward the dural attachments of the tumor. Devascularization of the tumor is performed with bipolar coagulation of the whole dural attachment, taking care to preserve the surrounding structures.

**Figure 1:**
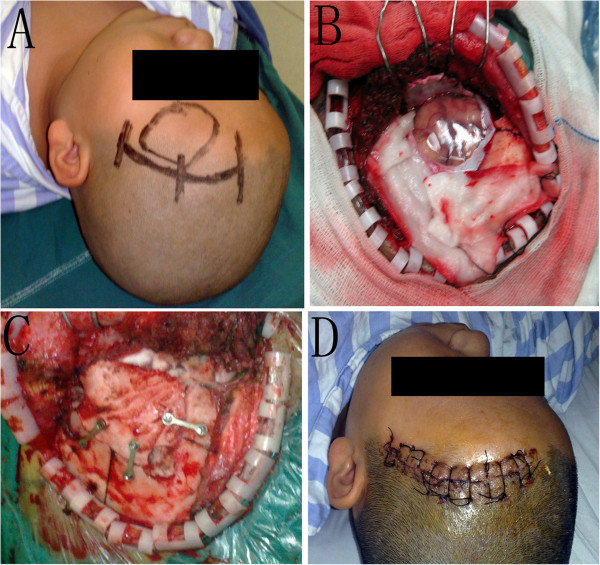
(A) the operative incision of LSO; (B) the dura was opened; (C) skull reconstruction; (D) suturation after LSO surgery.

The classic LSO approach has been described in detail in Hernesniemi’s reports [7]. In the present study, it was applied in 17 cases (74%). In the classic LSO approach, the Sylvian fissure remains at the inferior border of the dural opening and, if needed, it can be easily opened. In six cases (26%) we performed a LSO approach with temporal extension, that is, the classic LSO approach with additional lateral extension toward the middle cranial fossa for a minimal temporal exposure. This means extending the exposure for approximately 1 cm or so to the temporal side of the Sylvian fissure. The head position is the same as in a classic LSO approach but with a more caudal and posterior skin-muscle cut. The size of the tumor with a significant temporal component is the only indication for temporal extension of the LSO approach (Figure 1).

## Results

### LSO approach applied to pituitary adenoma

**Figure 2:**
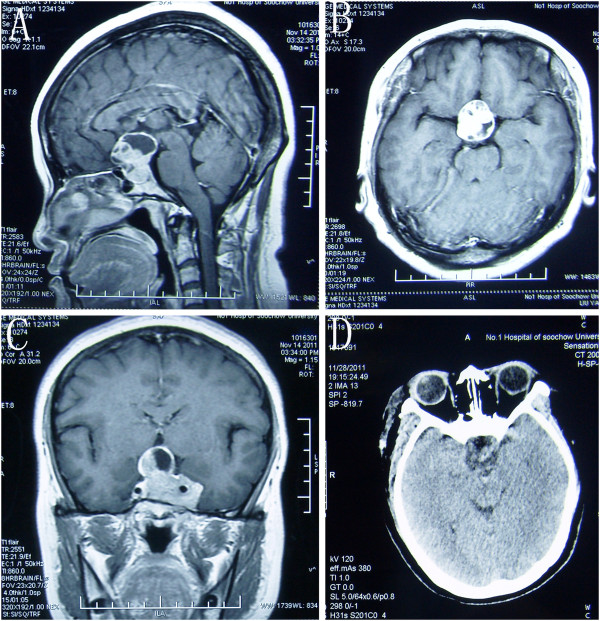
**Case number 9: Sagittal (A), axial (B), and coronal (C) view of contrasted magnetic resonance imaging showing a pituitary adenoma. (D)** Postoperative CT imaging of the same patient.

**Figure 3:**
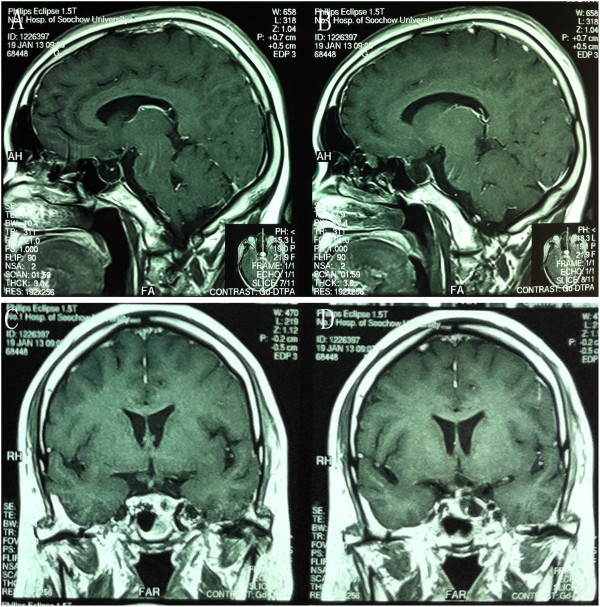
Case number 10: Axial (A-B) and coronal (C-D) view of contrasted magnetic resonance imaging showing total removal of pituitary adenoma.

Of the eleven cases, none (0%) were large (> 6 cm), nine (82%) medium (3 to 6 cm), and two (18%) small (< 3 cm). The average tumor size was 3.3 cm (range, 2.7 to 4.2 cm). Most cases (n = 7, 64%) were removed via a right (nondominant) LSO approach, which is more convenient for a right-handed neurosurgeon even if the tumor extends more to the left side. In four patients (36%), the approach was from the left, because of marked tumor extension to the left. As shown in Figure 2 (case number 9) and Table 1, total removal of the tumor was achieved in eight (73%) of eleven patients. The Karnofsky score at discharge was 80 to 100 and the GOS was 4 to 5, which meant the outcome was relatively good. Figure 3 shows the post-operative MR images of case number 10, which demonstrates that the tumor had been totally removed.

### LSO approach applied to craniopharyngioma

**Figure 4:**
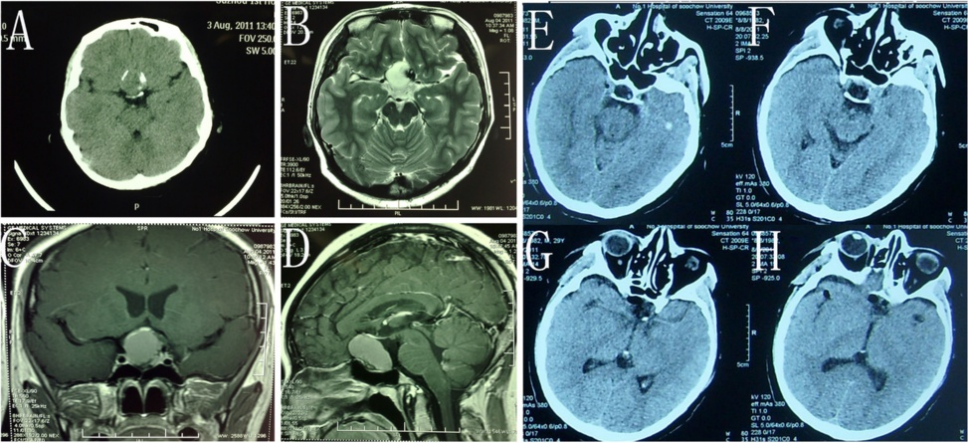
Case number 12: (A) preoperative CT imaging showing the calcification of craniopharyngioma; (B-D) contrasted magnetic resonance imaging showing a craniopharyngioma; (E-H) postoperative CT imaging of the same patient.

**Figure 5:**
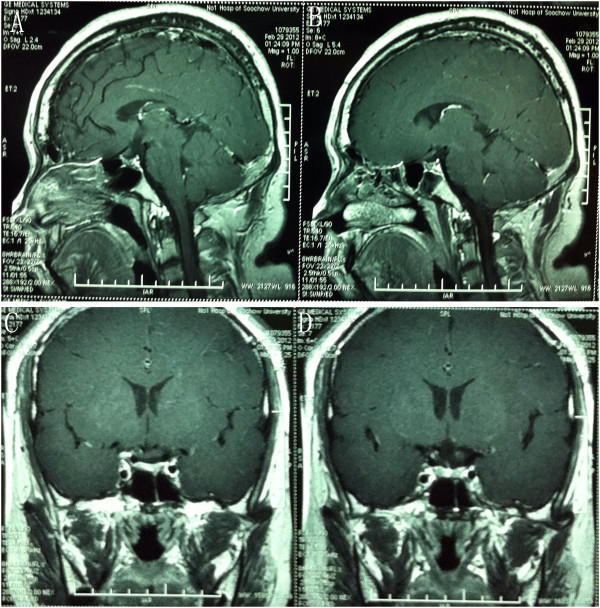
Case number 13: Axial (A-B) and coronal (C-D) view of contrasted magnetic resonance imaging showing total removal of craniopharyngioma.

As shown in Table 1, six patients underwent a supraorbital craniotomy. Maximal tumor size was 40 mm. Five (83%) had a major tumor component within the retrochiasmal space. Out of six patients, gross total, near total, and subtotal resection were achieved in two patients (33%), three patients (50%), and one patient (17%), respectively (Figure 4, case number 12). As shown in Table 1, preoperative visual loss resolved in three patients (50%) and was unchanged in two patients (33%) after LSO surgery. Four patients (one with subtotal resection and three with near total resection) received postoperative stereotactic radiotherapy and remained stable at last follow-up, ranging from 12 to 13 months. The Karnofsky score at discharge was 80 to 100 and the GOS was 4 to 5, which meant the outcome was relatively good. Figure 5 shows the post-operative MR images of case number 13, which demonstrates that the tumor had been totally removed.

### LSO approach applied to tuberculum sellae meningioma

**Figure 6:**
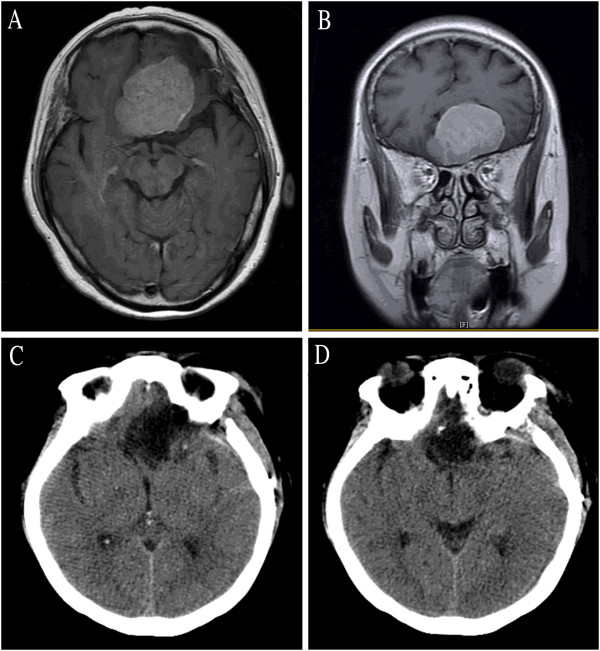
**Case number 20: Axial (A), and coronal (B) view of contrasted magnetic resonance imaging showing a tuberculum sellae meningioma. (C**-**D)** Postoperative CT imaging of the same patient.

**Figure 7:**
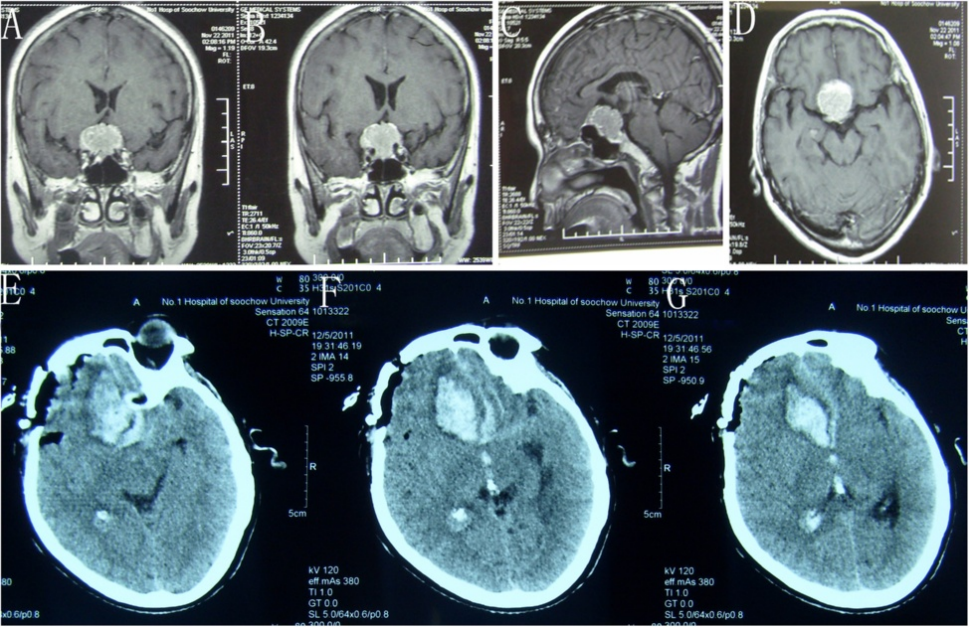
**Case number 22: (A-D) view of contrasted magnetic resonance imaging showing a tuberculum sellae meningioma.** (**E**-**G**) view of CT images showing a hematoma in the sellar region.

As shown in Table 1, six underwent supraorbital removal. Of patients undergoing LSO surgery, total/near total removal (> 90%) was achieved in six (100%) of six patients. Of patients treated by LSO approach removal, preoperative visual loss resolved in three (50%) of six patients, was unchanged in two patient (33%), and worsened in one (17%) of six patients (Table 1). Of six patients who had LSO meningioma removal, four had total removal of noninvasive tumors (Figure 6, case number 20), and none had tumor recurrence at a median follow-up of six months. The Karnofsky score at discharge was 0 to 100 and the GOS was 1 to 5, which meant the outcome was relatively acceptable. One patient died from postoperative bleeding and one patient’s GOS was 3 at discharge because of infarction in the sellar region. Figure 7 showed the preoperative MR images and postoperative CT images of case number 22, which was the case who died, and revealed a hemorrhage of the sellar region.

### LSO approach applied to a rare case (intracranial optic nerve Schwannoma)

**Figure 8:**
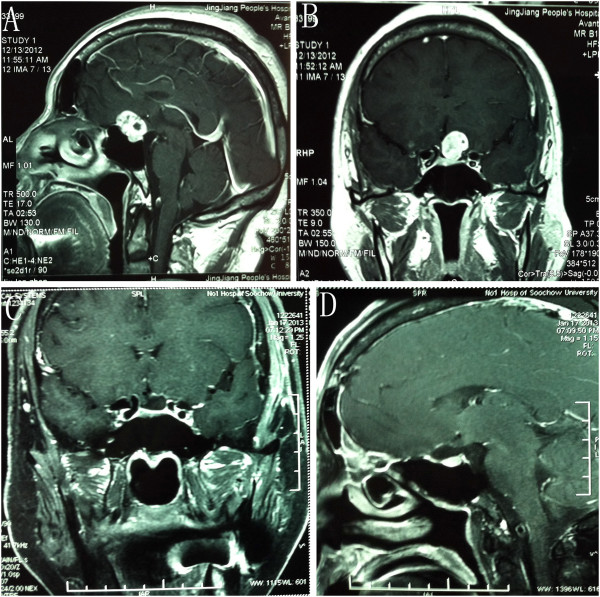
(A-B), preoperative MRI showing an intracranial optic nerve Schwannoma; (C-D) view of contrasted magnetic resonance imaging showing total removal of tumor.

The patient was a 57-year-old female who presented with complaints of increasingly impaired vision of the left eye. Neurological exam revealed mild impaired vision of left eye, but was otherwise unremarkable. MRI scan shows an approximately 2.5 cm well-demarcated lesion in the left superior sellar region (Figure 8). We performed a LSO approach for this patient, and the surgery was all right, in which we can see the tumor was from the left foramen opticum and was totally removed after opening the foramen opticum. The pathological diagnosis was Schwannoma and immunohistochemical testing showed Vimentin (+), S-100 (+), MBP (+), CD56 (+), GFAP (+), Ki-67 (+), Desmin (−), SMA (−), EMA (−), CD34 (positive in tumor vessels). The postoperative MR images are shown in Figure 8, and demonstrate that the tumor had been totally removed.

## Discussion

In this article, we have analyzed the clinical outcome and surgical complications of our recent consecutive series of 23 patients who have undergone surgery through the LSO approach for the treatment of neoplastic lesions in the sellar region. This was with the aim of 1) comparing the outcome of our series with other published series using different (generally more extensive) approaches and 2) understanding the technical nuances mostly affecting outcome; because of the vicinity of the optic nerve to the tumor, visual outcome is the most pertinent issue in this regard.

The most frequent approach for sellar tumors reported in the literature is the pterional one [15]. The microsurgical technique for sellar tumors through a pterional approach for the treatment of pituitary adenoma has been described in detail by other colleagues [16]. The standard pterional approach has been accepted as the gold standard in order to gain strategic access to a range of neurosurgical intracranial lesions [17]. Yasargil *et al*. [18] advocate this approach to achieve surgical control of the anterior circulation as well as basilar tip aneurysms. Although it is the preferred approach to treat various intracranial lesions, the standard pterional approach has been recently re-evaluated critically and compared with other approaches such as the supraorbital eyebrow incision approach, the mini-pterional approach, the mini-supraorbital approach, and the LSO approach [8]. Knowing the limitations of these procedures helps to determine the correct indications for each procedure, and using the advantageous features of these approaches will help to deliver the treatment that is the most appropriate for each individual patient.

Recently, previous reports have shown the surgical experience of Juha Hernesniemi in operating on olfactory groove [12], anterior clinoidal [14], and tuberculum sellae meningiomas through a LSO approach [6]. Hernesniemi has used the LSO approach instead of the pterional one for the past 25 years (Table 2). In larger tumors or giant aneurysms involving the medial sphenoid wing, the LSO approach can be modified with a 1 cm temporal extension to fully visualize the Sylvian fissure with its temporal portion [6]. The LSO approach is less traumatic, much faster, and simpler than the classic pterional approach and allows the surgeon to easily reach the sellar region [7]. The orbitozygomatic approach, also widely used and described in the context of sellar surgery in the literature [19] is, in our opinion, even more traumatic than the pterional one and needlessly extensive.

**Table 2:** Summary of the previous clinical reports on the LSO approach

**Year**	**Operator**	**Institute**	**Disease**	**Case SUM**	**Follow-up time**	**Outcome**
2012 [6]	Juha Hernesniemi	Helsinki University Central Hospital	Tuberculum sellae meningiomas	52	59 Months	Good
2012 [7]	Juha Hernesniemi	Helsinki University Central Hospital	Aneurysm, tumor, CCF	82	Not reported	Good
2012 [14]	Juha Hernesniemi	Helsinki University Central Hospital	Aneurysm, tumor, CCF	82	Not reported	Good
2011 [13]	Juha Hernesniemi	Helsinki University Central Hospital	Meningioma	191	Not reported	Good
			Anterior clinoidal			
2011 [20]	Juha Hernesniemi	Helsinki University Central Hospital	meningiomas	73	36 Months	Good
			Olfactory groove			
2009 [12]	Juha Hernesniemi	Helsinki University Central Hospital	meningiomas	66	45 Months	Good
2005 [11]	Juha Hernesniemi	Helsinki University Central Hospital	Sellar lesions	> 2000	Not reported	Good

The only experimental study on the LSO approach not conducted by Hernesniemi’s team was published in 2010 by Dr. Asem Salma from the Ohio State University Medical Center [8]. In that research, the authors provided a qualitative and quantitative anatomical comparison of the surgical exposure and the operability afforded by the standard pterional approach and the LSO approach. From an anatomical point of view, both approaches provide similar exposure to the sellar, suprasellar, and anterior communicating artery areas. The pterional approach provides better exposure of the retrosellar area. The ability to operate in the retrosellar area, as judged by their model, was higher with the pterional than with the LSO approach. On the basis of that study, the LSO approach would be anatomically indicated mainly for treating sellar and suprasellar lesions and anterior communicating artery aneurysms, where it could be used as a less invasive approach to these areas than the standard pterional approach. However, the standard pterional approach, by virtue of the shape and volume of its surgical space, could offer greater overall theoretical surgical operability. Hence, the standard pterional approach is anatomically suited for complex lesions of the sellar and parasellar spaces, especially when these lesions involve the interpeduncular cistern. Even with the intrinsic limitations of a cadaver environment, the results of our study highlight differences between the two approaches considered. In addition, our study proposes a helpful methodology to describe and contrast microneurosurgical approaches in a laboratory environment.

For the case of the patient who died, post-CT scanning demonstrated a hemorrhage of the sellar region. Following rapid surgical re-exploration, the patient initially survived, but after this second operation, complications including coagulation disorder, coma, hyperpyrexia, and electrolyte disturbances developed and three days after the surgery, the patient passed away. For LSO surgery, we should try our best to release CSF from the lateral fissure cistern, suprasellar cistern, and chiasmatic cistern to help decrease the pressure on the brain.

During the whole surgical process, neurosurgeons should try to prevent brain contusion caused by the brain spatula by keeping their hands light and soft, and performing the operations through the crevice of brain under the microscope. Sufficient understanding of the anatomical structures in the sellar region is a necessary condition for neurosurgeons to perform the LSO approach. In pituitary adenoma cases, we always found poor development of the sphenoidal sinus and in some cases the space of the saddle area was very small. So for these patients, we will choose an LSO approach to remove the tumor. On the other hand, some pituitary adenomas were mainly located at the suprasellar region and exhibited the ‘hourglass sign’. For these patients, we previously chose to use trans-longitudinal fissure, subfrontal, or pterion approaches, which might cause more brain injury to the patients than the LSO approach.

## Conclusion

In summary, with the simple and fast LSO approach for surgery of sellar tumors, our surgical results and tumor recurrence rates at follow-up are similar to those obtained using more extensive and time-consuming approaches. The LSO approach can be used for sellar lesions of all sizes and has a relatively low morbidity and mortality. Based on our experience, we recommend the LSO approach for surgery of sellar tumors.

### Consent

Written informed consent was obtained from the patient for publication of this report and any accompanying images.

## Abbreviations

CSF: cerebrospinal fluid; CT: computed tomography; GOS: Glasgow outcome scale; LSO: lateral supraorbital; MRI: magnetic resonance imaging; PA: pituitary adenoma; RC: retrochiasmal; S: sellar; SS: suprasellar.

## Competing interests

The authors declare that they have no competing interests.

## Authors’ contributions

GC has contributed to the conception, design, and acquisition of information and has written this paper. ZW and DZ have contributed to the analysis and interpretation of data. All authors read and approved the final manuscript.
